# Supplementary material for: Renogrit attenuates Vancomycin-induced nephrotoxicity in human renal spheroids and in Sprague-Dawley rats by regulating kidney injury biomarkers and creatinine/urea clearance
Source: PLoS One. 2023 Nov 8;18(11):e0293605. doi: 10.1371/journal.pone.0293605 (PMC10631690; doi:10.1371/journal.pone.0293605)
Supplement: S1 Fig — (DOCX) [file pone.0293605.s001.docx]

Supporting information

Renogrit Attenuates Vancomycin-induced Nephrotoxicity in Human Renal Spheroids and in Sprague-Dawley Rats by Regulating Kidney Injury Biomarkers and Creatinine/Urea Clearance

Acharya Balkrishna, Sonam Sharma, Vivek Gohel, Ankita Kumari, Malini Rawat, Madhulina Maity, Sandeep Sinha, Rishabh Dev, Anurag Varshney^*^

*** Correspondence:** anurag@patanjali.res.in (AV)

**Supporting figure**


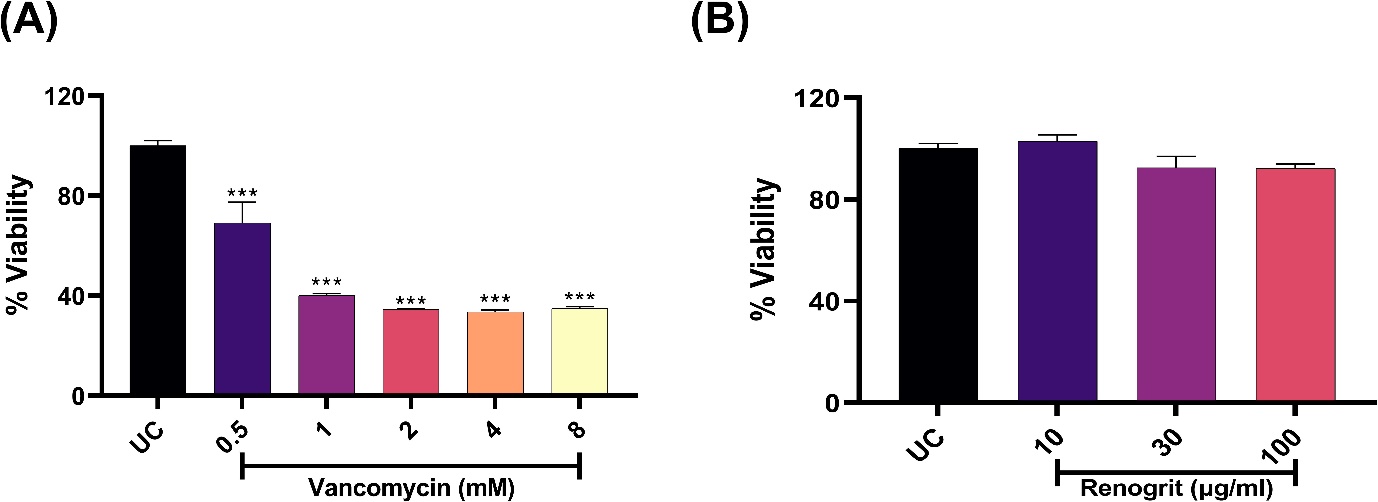


**Supporting Fig 1. Viability assessment. (A)** Vancomycin (0.5-8 mM) and **(B)** Renogrit (10, 30, and 100 µg/mL) on HK2 spheroids. Data represented as mean ± SEM (n=3). ***, *p* < 0.001 *vs*. Untreated control group.
