# Supplementary material for: Renogrit attenuates Vancomycin-induced nephrotoxicity in human renal spheroids and in Sprague-Dawley rats by regulating kidney injury biomarkers and creatinine/urea clearance
Source: PLoS One. 2023 Nov 8;18(11):e0293605. doi: 10.1371/journal.pone.0293605 (PMC10631690; doi:10.1371/journal.pone.0293605)
Supplement: Attachment — Submitted filename: Response to reviewer comments.docx [file pone.0293605.s003.docx]

**Reviewer 1**

**Comment:** The manuscript has been greatly improved based on the comments provided in the first revision. I have no further comments.

**Response:** We thank the reviewer for the comment.

**Reviewer 3**

**Comment:** The authors have answered the questions. Unfortunately, the answered comments are not marked with a yellow bar in the main text of the article. This can help the reviewer to check. The reference for all methods is not mentioned.

**Response:** We acknowledge the comment by the reviewer. The answered comments have now been marked with a yellow bar in the main text of the article. Also, additional references have been added in the methods section. As per the journal requirements, a clean copy of manuscript has to be provided, so highlighted version of the manuscript (with track changes) has been kept at the end of the clean copy of the manuscript.
